# Report of natural *Mayaro virus* infection in *Mansonia humeralis* (Dyar & Knab, Diptera: Culicidae)

**DOI:** 10.1186/s13071-023-05707-2

**Published:** 2023-04-24

**Authors:** Flávia Barreto De Sousa, Juliana Santana de Curcio, Lívia do Carmo Silva, Diego Michel Fernandes da Silva, Silvia Maria Salem-Izacc, Carlos Eduardo Anunciação, Bergmann Morais Ribeiro, Marco Tulio A. Garcia-Zapata, Elisângela de Paula Silveira-Lacerda

**Affiliations:** 1 Unidade Sentinela e Centro de Referência em Medicina Internacional e de Viagens (USCREMIVI)/Núcleo de Estudos e Pesquisa de Agentes (Re) Emergentes (NUPEREME), Universidade Federal de Goiás, IPTSP/ICB, Goiânia, Goiás Brazil; 2 Universidade de Brasília, Brasília, Distrito Federal Brazil

**Keywords:** Arboviruses, *Mansonia humeralis*, Mayaro fever, Viral isolation

## Abstract

**Background:**

Arboviruses are a group of viruses transmitted to vertebrate hosts by certain blood-feeding arthropods. Among urban vectors of arboviruses, mosquitoes of the genus *Aedes* are the most common. However, other mosquitoes may be susceptible to infection and involved in the transmission, such as *Mansonia* spp. Therefore, this study aimed to investigate whether *Mansonia humeralis* can be infected with the *Mayaro virus* (MAYV).

**Methods:**

These insects were collected from 2018 to 2020 in chicken coops of rural communities in Jaci Paraná in Porto Velho, Rondônia, Brazil, while performing blood-feeding on roosters. The mosquitoes were randomly grouped in pools from which the head and thorax were macerated and checked for the presence of MAYV by quantitative reverse transcription polymerase chain reaction (RT-qPCR). The positive pools were used to infect the C6/36 cell line, and on different days post-infection, the supernatant of the infected cells was subjected to viral detection by RT-qPCR.

**Results:**

A total of 183 pools of female mosquitoes were tested, of which 18% were positive for MAYV; some samples from insect pools inoculated into C6/36 cells showed in vitro multiplication capacity between 3 and 7 days post-infection.

**Conclusions:**

This is the first report of *Ma. humeralis* mosquitoes that are naturally infected by MAYV, indicating that these vectors may be potential transmitting agents of this arbovirus.

**Graphical Abstract:**

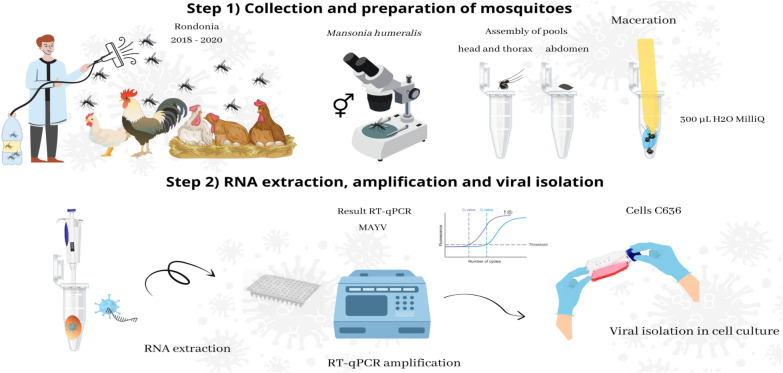

**Supplementary Information:**

The online version contains supplementary material available at 10.1186/s13071-023-05707-2.

## Background

Arboviruses, or viruses transmitted by arthropods, are potentially fatal and epidemic, representing a serious public health problem with social and economic implications. Except for yellow fever and dengue, no vaccines and effective antiviral drugs or therapy are available to treat arboviral diseases. Therefore, vector control is an essential tool for preventing future outbreaks. Furthermore, screening for arboviruses in mosquito species can provide information about which vectors are participating in the local dynamics of viruses [1].

The increase in the incidence of the *Mayaro virus* (MAYV; genus *Alphavirus*, family *Togaviridae*) has attracted the attention of researchers and public health authorities. MAYV was discovered in 1954 when it was isolated from the blood of febrile patients with arthralgia in Trinidad and Tobago [2]. It is endemic to South America and the Caribbean. Moreover, MAYV has been detected in Argentina, Bolivia, Brazil, Ecuador, French Guiana, Haiti, Mexico, Panama, Peru, and Venezuela. Currently, four distinct genotypes are in circulation. In Brazil, epidemiological studies have shown the presence of the virus in different regions, including the north, central-west, and southeast [3, 4].

Understanding the arthropod vectors that naturally transmit MAYV is critical to elucidating the interactions between biological and environmental factors that affect the transmission of this arbovirus and identifying regions of increased outbreak probability. The primary vector of MAYV is *Haemagogus janthinomys* [5]. However, other mosquito species have been described as susceptible to MAYV and thus can be competent in arbovirus transmission, such as *Psorophora ferox*, *Psorophora albipes*, *Sabethes* spp., *Culex* spp., and *Aedes* spp. [6, 7]. Aitken et al. [8] demonstrated the vector competence of the *Aedes scapularis* mosquito in transmitting MAYV by inoculating the virus into female mosquitoes and inducing them to feed on the blood of infected *Gallus gallus domesticus* and/or *Mus musculus*. This mechanism was additionally used to demonstrate that MAYV can also infect *Culex* spp. and *Anopheles* spp. [6, 7, 9, 10]. Recently, MAYV was detected in eggs and male mosquitoes of the genera *Aedes* and *Culex* in Mato Grosso, Brazil, suggesting vertical transmission [11–13]. De Curcio et al. [13] also demonstrated the possible vertical transmission of MAYV by *Aedes aegypti* in Goiás, Brazil. In addition, the genus *Mansonia* has been reported to be naturally infected with several arboviruses, such as MAYV [12], *Chikungunya virus* (CHIKV), and *Zika virus* (ZIKV) [14, 15]. However, *Mansonia* spp. mosquitoes are not considered a vector of endemic diseases in Brazil.

Recently, anthropogenic activities have led to significant ecological changes that create breeding grounds for vector proliferation and acquisition, posing a risk to human health [16, 17]. For example, Jaci Paraná, a district of Porto Velho, Rondônia, in northern Brazil, has several factors that favor the spread of vectors. This district has experienced several environmental changes, such as deforestation, rural settlements, and the establishment of extensive cattle ranching. These changes provided suitable areas for mosquito breeding, resulting in high population densities of vectors such as *Mansonia* spp. [18–20].

It is well known that early detection of arboviruses in mosquitoes can aid in effective decision-making about public health interventions to reduce the risk of outbreaks of these diseases. Therefore, this work describes the natural occurrence of MAYV in *Ma. humeralis* for the first time.

## Methods

### Collection of mosquitoes and processing of samples

Mosquitoes were collected from 2018 to 2020 from chicken coops while feeding on the blood of roosters (*G. gallus domesticus*) weighing more than 2 kg in rural communities near a hydroelectric power plant in Jaci Paraná, Porto Velho, Rondônia (RO), Brazil (latitude: −9.2654178 and longitude: −64.4008246). A manual vacuum cleaner was used to collect the mosquitoes. These mosquitoes were kept in the gut for 2 days for blood digestion and then *Ma. humeralis* were identified morphologically and kept at −80 °C.

A total of 1930 mosquitoes were grouped into 183 pools of female individuals and one pool of male individuals. Each pool contained 10 mosquitoes. The head and thorax of females were detached and processed, while males were processed intact. Briefly, mosquitoes were macerated in 300 µl of sterile Milli-Q water in 1.5-ml centrifuge tubes (Axygen, USA) using a pestle. The macerated mosquitoes were centrifuged at 10,000×*g* for 2 min, and the supernatant was then collected and stored at −80 °C until viral RNA extraction. RNA extraction was performed using the QuickExtract™ RNA Extraction Solution (Lucigen, Miami, FL, USA) kit according to the manufacturer's instructions. All reagents used for mosquito manipulation were tested in quantitative reverse transcriptase polymerase chain reaction (RT-qPCR) to exclude any internal contamination.

### Molecular detection of MAYV

The presence of MAYV in the RNA samples was detected by RT-qPCR with the AriaMX system (Agilent Technologies, Santa Clara, CA, USA) using the GoTaq^®^ Probe 1-Step RT-qPCR System Kit (Promega, Madison, WI, USA) according to the manufacturer's recommendations. The set of primers/probes amplifying the actin gene of *Mansonia* spp. were used as an endogenous control of RT-qPCR. We used gBlocks (Integrated DNA Technologies, Coralville, IA, USA), recognized by the primer and probe pairs designed to amplify the MAYV nonstructural polyprotein gene (GenBank: MK956954.1) or the actin gene of *Mansonia* spp. (GenBank: GQ981455.1) as a positive control. The viral genome copy number was calculated by plotting the quantification cycle (C_q_) values reported in RT-qPCR against the standard curve [13] in which the threshold for detection of endogenous control (actin) and MAYV was determined, and C_q_ values above the detection threshold were considered negative. The gBlocks, probes, and primer sequences used in this study are listed in Additional file 1: Table S1 and Additional file 2: Table S2, respectively. The detection limit for the actin gene was five copies with a C_q_ value of 34.30 (Additional file 3: Fig. S1). For MAYV, the detection limit was 10 copies with a C_q_ value of 34.98 [13].

### Inoculation of positive pools in cell culture

A total of four positive pools in the RT-qPCR analysis were used in the experiments in cell culture to confirm viral replication. The *Aedes albopictus* clone C6/36 cells (American Type Culture Collection [ATCC] CRL-1660™) were grown in TC100 medium (Vitrocell, São Paulo, Brazil) supplemented with 10% fetal bovine serum (FBS; LGC Biotechnology) at 28 °C. After 70–80% cell confluence, 100 µl of macerated supernatant from mosquito pools was filtered through a 0.22-μm sterilization membrane (Nalgene-Thermo Scientific, Waltham, MA, USA) and inoculated into C6/36 cells. Uninfected cells were used as negative controls. Cells were then incubated at 28 °C, and cell morphology was observed every 24 h for 7 days using an inverted phase-contrast microscope (Leica, DMi1). After 3 and 7 days, 2 ml of the culture supernatant was collected, viral RNA was extracted, and a new RT-qPCR was performed with these samples, as described in the “*Mayaro virus* recovery in the culture supernatant” section.

### Data analysis

RT-qPCR results were analyzed using Agilent AriaMx software (version 1.8; Agilent Technologies, Santa Clara, CA, USA). Excel software was used to construct standard curves and determine the straight line of the equation, tables, and graphs. The minimum infection rate (MIR) was calculated using the formula [(number of positive pools/total specimens of the species tested) × 1000] [21] and reported with a 95% confidence interval (EpiData Analysis, 2006–2010).

## Results

### Mosquito screening and molecular detection of MAYV

A total of seven mosquito species of the genus *Mansonia* were collected and identified, including *Mansonia venezuelensis*, *Ma. flaveola*, *Ma. pseudotitillans*, *Ma. amazonensis*, *Ma. titillans*, *Ma. wilsoni*, and *Ma. humeralis.* *Ma. humeralis* was the most abundant species and was therefore selected for molecular analysis.

A total of 1930 *Ma. humeralis* were screened for MAYV using RT-qPCR. MAYV genetic material was detected in 34 (18%) female pools (Table 1). These data correspond to an MIR value of 19. Table 1 lists the C_q_ values ​​obtained and their equivalent copy number. The C_q_ values ranged from 25.75 to 34.74, corresponding to a variation of 2287 to 9 viral copies/µl. Viral RNA was not identified in the male pool (data not shown).

**Table 1:** Detection of *Mayaro virus* using RT-qPCR in pools of female *Mansonia humeralis*

RT-qPCR MAYV			RT-qPCR MAYV		
Pool number	C_q_	Number of copies/µl	Pool number	C_q_	Number of copies/µl
2	34.47	10	25	33.82	16
3	33.45	19	26	33.61	18
4	31.59	61	28	27.39	821
5	33.05	25	30	30.74	104
6	32.90	27	31^b^	28.69	368
8	29.30	253	32	33.43	20
10	32.69	31	34	33.40	20
12	31.58	62	41	31.19	79
14	30.45	124	42	31.24	76
15	33.92	15	43^c^	29.14	279
18	34.71	9	45	34.74	9
19	34.53	10	46^d^	28.88	327
20	30.90	94	48	25.73	2287
21	32.12	44	49	34.41	11
22^a^	26.27	1639	50	30.71	106
23	30.26	140	57	34.58	10
24	33.02	25	184	26.82	1167

^a–d^These pools were used for the in vitro infection experiment

### *Mayaro virus* recovery in the culture supernatant

**Fig. 1:**
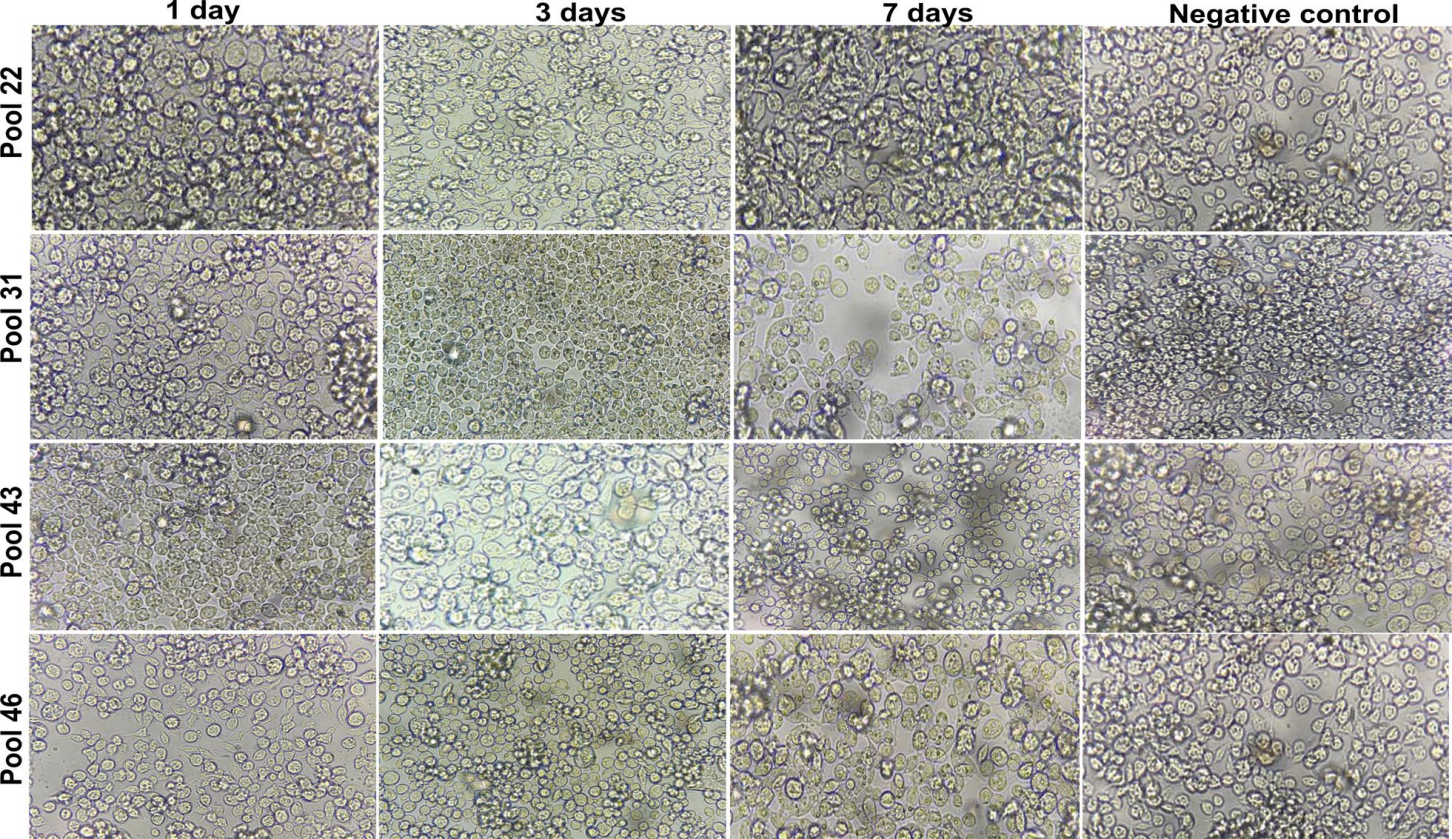
Monitoring of the effect of MAYV inoculation in C6/36 cells after 1, 3, and 7 days. The images were analyzed by phase-contrast microscopy, with ×40 magnification. The culture supernatant at these time points was collected and analyzed by RT-qPCR

To evaluate the replication of MAYV in cell cultures, the pools with the highest viral load (22, 31, 43, and 46 samples) were inoculated into C6/36 cells. Morphological analysis showed no cytopathic effect in the cells inoculated with MAYV. However, molecular analysis showed the presence of the viral material in the cell culture. Images of the infected cells are shown in Fig. 1. All MAYV pools inoculated into C6/36 cells survived, as evidenced by the presence of the viral RNA in the culture supernatant. Replication was less evident in pools 22, 31, and 43 during infection. Otherwise, pool 46 showed remarkable viral replication capacity, as indicated by the number of viral copies/µl detected after 7 days of cultivation (Table 2). These results suggest that MAYV is present in *Ma. humeralis* and can survive and replicate in other cell models.

**Table 2:** C_q_ values and estimated number of viral copies/µl 3 and 7 days after infection of C6/36 with MAYV

Pool number	Infection day	C_q_	Number of copies/µl
22	Macerated	26.27	1639
	3 days	32.48	35
	7 days	32.74	30
31	Macerated	29.69	198
	3 days	29.95	169
	7 days	30.07	157
43	Macerated	29.14	279
	3 days	32.14	44
	7 days	33.94	15
46	Macerated	28.98	327
	3 days	31.87	52
	7 days	28.63	382

## Discussion

Hundreds of arboviruses are known so far, and several species can affect domestic and wild animals [22]. However, approximately 134 have been reported to cause human diseases [23]. The main vectors of arboviruses in urban areas are *Ae. aegypti* and *Ae. albopictus*; however, other vectors are also involved in the transmission of arboviruses in urban and wild cycles. It is estimated that such arboviruses are responsible for 390 million infections annually [24]. Due to the presence of humans in forested regions and the adaptation of mosquitoes to urban areas, new vectors of arboviruses are being identified. In this sense, at least six viral RNA families and several unclassified viruses were detected in *Ma. wilsoni* collected in the Atlantic Forest in the northeast region of Brazil [25]. Analysis performed in the last decade indicates that novel mosquito-associated viruses primarily infect the *Culex*, *Anopheles*, *Aedes*, and *Mansonia* genera [26].

*Mansonia* spp. are voracious hematophagous mosquitoes whose mature stages usually breed in freshwater bodies with aquatic vegetation. Reductions in water flow lead to the proliferation of aquatic plants, increasing their populations. The dispersal of insect vectors is significant for epidemiology, as dispersal is a crucial factor in the course of disease outbreaks and the population dynamics of vector and pest arthropods [27, 28]. Jaci Paraná, Porto Velho, Brazil, is an area that has undergone several environmental changes due to deforestation, the creation of rural settlements, and the establishment of an extensive beef cattle system. These changes led to new areas with suitable vegetation for insect breeding and a high food supply for *Mansonia* spp. females to perform hematophagy, resulting in a high population density of *Ma. humeralis* in this region [25]. Nonhuman vertebrates such as chickens [29, 30] also contribute to the persistence of these viruses, as they usually serve as reservoirs for various arboviruses. In this work, an increase in the population of *Mansonia* spp. which fed on the blood of these animals was observed in chicken coops in Jáci Paraná. Therefore, all these processes may contribute to MAYV spreading to other hosts in nature, as is the case with other viruses such as SARS-CoV-2.

The significant increase in vector population and genetic variability allows the emergence of populations with traits that favor susceptibility to infection and make them competent to transmit different arboviruses [31]. *Ma. venezuelensis* and *Ma. uniformis* have previously been reported to be naturally infected with the arboviruses MAYV, CHIKV, and ZIKV [14, 15, 32], indicating that they are potential vectors. However, more information about their vector competence is needed. In this work, we detected the presence of MAYV viral RNA in 18% of *Ma. humeralis* pools collected in Jaci-Paraná district. In addition, the virus replication in cell cultures was observed in four positive pools.

The presence of MAYV in these mosquitoes indicates the occurrence of natural infection, since most of the positive pools were not engorged. Therefore, the detected virus was not acquired from vertebrate host blood. However, the isolation of MAYV from other Culicidae species, such as *Aedes*, *Culex*, *Psorophora*, *Sabethes*, and *Haemagogus* spp., has been described [33, 34].

MAYV is endemic to the Amazon region. An epidemiological study between 1955 and 2019 reported 1304 cases of MAYV infection in Brazil, with 1142 cases described only in the northern region [4], which includes the states of Pará, Amazonas [35], and Tocantins [36]. *Ma. humeralis* can travel distances greater than 2000 m from the site of adult emergence. In addition, Mello et al. [19] describe the occurrence of passive dispersion that occurs through floating aquatic macrophytes that, once containing immature species of *Mansonia* spp., are often dragged by river currents, favoring the distribution of these mosquitoes to different locations [18].

The increase in the population and spread of arbovirus vectors due to anthropogenic impacts could increase the transmission of emerging and re-emerging arboviruses. In this sense, the data presented in this work suggest a possible involvement of *Ma. humeralis* in the epidemiological cycle of MAYV in Jáci-Paraná; however, further studies are needed to clarify this mechanism.

## Conclusions

This is the first report of *Ma. humeralis* mosquitoes naturally infected with MAYV. The detection of MAYV in the head and thorax pools and its replication in cell culture suggest the susceptibility of these mosquitoes to infection with this virus. In addition, these mosquitoes may be involved in the transmission of these arboviruses in the district of Jaci Paraná, Rondônia, Brazil. Therefore, we emphasize the need for greater attention from public health authorities and the development of effective control measures for the mosquito population in Jaci Paraná (RO) district.

## Supplementary Information

**Additional file 1: Table S1**. gBlocks sequence used for RT-qPCR tests.

**Additional file 2: Table S2**. The sequence of primers to identify arboviruses and actin of insect vectors *Aedes *spp., *Mansonia* spp., and *Culex* spp.

**Additional file 3: Fig. S1.** Standard curves to identify the limit of detection of actin gene through the real-time PCR technique. The log^10^ dilution series, ranging from 10^5^ to 1 copy of the amplicon of the actin gene in the block/reaction, was used to build the standard curve; the correlation coefficient (*R*^2^) values were above 0.99.

## Data Availability

Not applicable.

## Abbreviations

MAYV: *Mayaro virus*
RT-qPCR: Reverse transcription-quantitative polymerase chain reaction
