# Supplementary material for: Report of natural Mayaro virus infection in Mansonia humeralis (Dyar & Knab, Diptera: Culicidae)
Source: Parasit Vectors. 2023 Apr 24;16:140. doi: 10.1186/s13071-023-05707-2 (PMC10124708; doi:10.1186/s13071-023-05707-2)
Supplement: Supplementary file 1 — Additional file 1: Table S1. gBlocks sequence used for RT-qPCR tests. [file 13071_2023_5707_MOESM1_ESM.docx]

**Additional file 1:** **Table S1.** Gblock sequence used for RT-qPCR tests.

| **Gblock OROV e ACT (Oropouche and Actin)* and Gblock ZDCM (Zika, Dengue, Chikungunya, Mayaro)** |
| --- |
| 1- 5’GACCGACTACCTGATGAAGATCCTGACCGAGCGTGGCTACTCCTTCACCACTACCGCTGAACGTGAAATCGTTCGTGACATTAAGGAGAAGCTGTGCTACGTCGCTCTGGACTTCGAGCAGGAAATGGCCACCGCTGCTGCTTCCACCTCCCTGGAGAAGTCTTATGAACTTTTGACAAGTGCTCAATGCTGGTGTTGTTAGAGTCTTCTTCCTCAACCAAAAGAAGGCCAAAGATGTCTTACGTAAGACATCGAGGCCCATGGTTGACCTTACTTTTGGTGGGGTCCAATTTGCAATGGTTAATAACCATTTCCCACAGTTCCAGTCGAATCCAGTGCCGGACAACG3’  2-  5’CCGCTGCCCAACACAAGGTGAAGCCTACCTTGACAAGCAATCAGACACTCAATATGTCTGCAAAAGAACGTTAGTGGTTTTGGTTAGAGGAGACCCCTCCCTTACAAATCGCAGCAACAATGGGGGCCCAAGGTGAGATGAAGCTGTAGTCTCACTGGAAGGACTAGAGGTTAGAGGAGACCCCCCCGAAATAAAAAACAGCATATTGACGCTGGGAAAGACCAGAGATCCTGCTGTCTCTTTTAAAGGGCAAACTCAGCTTCACATGCCGCTGTGATACAGTGGTTTCTTTTGTGCGAGGGCTACGTCGTTAAGAGAATAACGATGAGCCCAGGCTTTTATAGACGACCTGCAGTCAGTGATGGCGACCCCGGACAATGAGTCACGGACATTTTGCCTTCACACAGATCAGACATGCAGGACTCCAGCTGAGGTGGCAGTCTATCA3’ |

* The underlined line represents the region for the actin (1) and MAYV (2) amplicon, respectively. In the design of the gblocks, sequences for other genes were included but not used in this study.
