# Supplementary material for: Report of natural Mayaro virus infection in Mansonia humeralis (Dyar & Knab, Diptera: Culicidae)
Source: Parasit Vectors. 2023 Apr 24;16:140. doi: 10.1186/s13071-023-05707-2 (PMC10124708; doi:10.1186/s13071-023-05707-2)
Supplement: Supplementary file 2 — Additional file 2: Table S2. The sequence of primers to identify arboviruses and actin of insect vectors Aedes spp., Mansonia spp., and Culex spp. [file 13071_2023_5707_MOESM2_ESM.docx]

**Additional file 2:** **Table S2.** The sequence of primers to identify arboviruses and actin of insect vectors *Aedes* sp., *Mansonia* sp. And *Culex* sp.

| **Target** | **Sequence forward** | **Sequence reverse** | **Probe** |
| --- | --- | --- | --- |
| Actin mosquitoes (^a^) | 5’GACYGACTACCTGATGAAGATC 3’ | 5’GTTCATAAGACTTCTCCAGGG 3’ | 5’ SUNCTGGACTTCGAGCAGGAAATGG 3’ IowaBlack |
| MAYV(^b^) | 5’-ATAGACGACCTGCAGTC-3’ | 5’TGATAGACTGCCACCTC-3’ | 5’56FAM/TCCTGCATGTCTGATCTGTGTGAAGGC/3iABkFQ/3’ |

(^a^) The oligonucleotide used in this work was acquired by IDT (Integrated DNA Technologies) and designed by the research group.

(^b^) The oligonucleotide used in this work was acquired by IDT (Integrated DNA Technologies) as described by the centers for disease control and prevention CDC.
