# Supplementary material for: Report of natural Mayaro virus infection in Mansonia humeralis (Dyar & Knab, Diptera: Culicidae)
Source: Parasit Vectors. 2023 Apr 24;16:140. doi: 10.1186/s13071-023-05707-2 (PMC10124708; doi:10.1186/s13071-023-05707-2)
Supplement: Supplementary file 3 — Additional file 3: Fig. S1. Standard curves to identify the limit of detection of actin gene through the real-time PCR technique. The log10 dilution series, ranging from 105 to 1 copy of the amplicon of the actin gene in the block/reaction, was used to build the standard curve; the correlation coefficient (R2) values were above 0.99. [file 13071_2023_5707_MOESM3_ESM.docx]

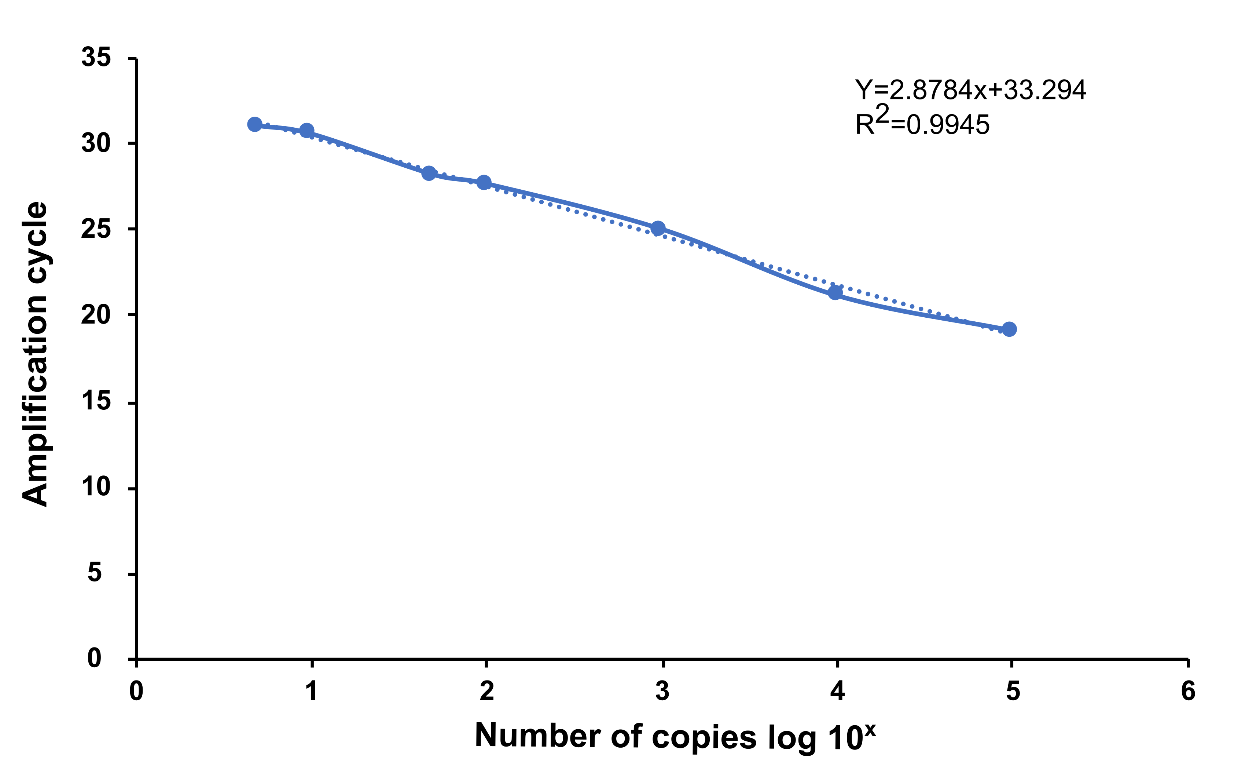


**Additional file 3: Fig.S1.** Standard curves to identify the limit of detection of actin gene through the real-time PCR technique. The log^10^ dilution series, ranging from 10^5^ to 1 copy of the amplicon of the actin gene in the block/reaction, was used to build the standard curve; the correlation coefficient (R^2^) values were above 0.99.
